# Six weeks of isometric resistance training led to evidence of corticospinal but not reticulospinal adaptation in previously untrained adult males

**DOI:** 10.1007/s00221-026-07391-x

**Published:** 2026-09-03

**Authors:** Simon Walker, Katherine E. J. Keogh, Meghan Tanel, Aidan S. W. Baker, Anne M. E. Baker, Dawson J. Kidgell, Stuart N. Baker

**Affiliations:** 1 NeuroMuscular Research Center, Faculty of Sport and Health Sciences, University of Jyväskylä, Jyväskylä, Finland; 2 Institute of Biosciences, Faculty of Medical Sciences, Newcastle University, Newcastle upon Tyne, UK; 3 School of Sport, Exercise and Health Sciences, Loughborough University, Loughborough, UK; 4 Department of Computing, Imperial College London, London, UK; 5 Monash Exercise Neuroplasticity Research Unit, Department of Physiotherapy, School of Primary and Allied Health Care, Faculty of Medicine, Nursing and Health Science, Monash University, Melbourne, Australia

**Keywords:** Strength training, Rate of force development, Startle, Transcranial magnetic stimulation

## Abstract

The latest hypothesis regarding the source of enhanced neural activation from resistance training is the reticulospinal rather than the corticospinal tract, based on invasive animal and emerging human data. The present study employed a six-week isometric resistance training intervention in a randomized controlled design to address this knowledge gap. Thirty-nine healthy, untrained males (age ~23 y, sustained contraction group n = 13, explosive contraction group n = 9, control group n = 17) underwent neuromuscular and electrophysiological testing and completed all study requirements. Maximal isometric torque (MVC) and rate of torque development (RTD) were measured during a familiarization session as well as before and after the six-week period. Transcranial magnetic stimulation was used to assess motor-evoked potential (MEP) area and silent period duration while subjects contracted to 10% of MVC. Loud sound (120 dB) was used to modulate MEP area and reaction time to visual stimuli during the StartReact test. Only the intervention groups demonstrated significant improvements in MVC (27%) and RTD (60%) (both P < 0.01), along with reduced MEP area (− 21%) and silent period duration (− 23%) (both P < 0.01). The sustained contraction group showed reduced modulation of reaction time and increased MEP suppression due to loud sound. Short-term resistance training seemed to reduce cortical inhibition and corticospinal excitability in both training groups. The study showed conflicting changes in measures purported to evaluate reticulospinal functioning. It is recommended to examine different forms of resistance training and longer training exposure in future.

## Introduction

It is well known that resistance training leads to early increases in maximal force production capacity, i.e. strength. Even repeated strength testing in naïve persons, without training per se, leads to increased force (Christie and Kamen 2010; Ploutz-Snyder and Giamis 2001). Such early increases in strength have long been accepted as having a neural origin (Moritani and DeVries 1979). Neuromuscular research has provided good evidence that the final neural input to skeletal muscle, motor unit behavior, adapts to enhance recruitment and discharge rate during force production tasks (Del Vecchio et al. 2019; Van Cutsem et al. 1998). However, the upstream neural mechanism(s) that contribute(s) to such changes in motor unit behavior have not been identified.

Descending pathways from the primary motor cortex (M1) to muscles have been investigated using transcranial electrical stimulation (Carroll et al. 2002) and transcranial magnetic stimulation (Nuzzo et al. 2017). Such stimulation leads to a motor-evoked potential (MEP) in the surface electromyogram of the contralateral muscle, where its amplitude is dependent on the number of excited neurons and subsequent modulation (i.e. facilitation and suppression) along the motor pathway. These methods can be applied either in a resting state or in conjunction with a (typically submaximal) voluntary contraction. Compared to magnetic stimulation, electrical stimulation is associated with greater discomfort, which has contributed to a preference for transcranial magnetic stimulation (TMS). From resistance training intervention studies employing TMS, meta-analyses have demonstrated a clear reduction in inhibitory mechanisms, purportedly within the M1, while yielding moderate and conflicting evidence for changes in MEP amplitude (Kidgell et al. 2017; Siddique et al. 2020a).

The MEP elicited by TMS reflects the summated contribution of direct (D−) and indirect (I−) waves, which arise from the activation and propagation of distinct corticospinal pathways (Sakai et al. 1997; Siebner et al. 2022). The latency of successive I-waves is considered indicative of the number of synaptic relays involved in corticospinal transmission. Therefore, although examining MEPs induced by contralateral TMS is thought of as a test of corticospinal functioning, different motor pathway contributions, including subcortical (e.g. reticulospinal tract), cannot be discounted.

Invasive research in monkeys has shown that total discharge of reticulospinal neurons increases with increasing external load while corticospinal neuronal discharge remains unchanged (Glover and Baker 2022). These findings have been corroborated in humans using functional magnetic resonance imaging (Danielson et al. 2024), showing that the BOLD signal within the pontine reticular nuclei increased in parallel with voluntary force output whereas the BOLD signal from the contralateral motor cortex did not. Additionally, daily training with increasing external load for 40 days led to increased spinal gain following reticulospinal tract stimulation with no changes in response to corticospinal tract stimulation in monkeys (Glover and Baker 2020). Thus, greater emphasis on methods that assess reticulospinal tract function may improve understanding of the neural mechanisms underpinning strength adaptations in humans following resistance training interventions, with one recent study supporting such adaptation (Akalu et al. 2026).

One validated method (Tapia et al. 2022) to probe and examine the reticulospinal tract is to utilize loud sounds to facilitate discharges from the reticular formation, located in the medulla, pons and midbrain. The StartReact paradigm demonstrates that pairing a loud auditory stimulus with a visual reaction task reduces reaction time and increases the rate of force/torque development, motor unit discharge rate (Skarabot et al. 2022), and muscle activity (Walker et al. 2024). Pairing loud sounds with a rapid force production test leads to enhanced rapid force generation or power performance (Fernandez-Del-Olmo et al. 2014). The acute modulations induced by the loud sound mirror increased motor unit discharge rate and maximal performance adaptations observed following resistance training interventions (Del Vecchio et al. 2019; Häkkinen and Komi 1981; Van Cutsem et al. 1998). Additionally, loud sound has been shown to reduce electromechanical delay during a dynamic reaction task (Eilfort and Filli 2025), which relates to the rate of force transmission through the tendon. Therefore, to perform fast contractions maximally, discharging motor units optimally and leveraging force transmission of series elastic elements, performance may be dependent on the efficacy of the reticulospinal tract.

TMS can also be paired with loud sounds at various latencies to suppress MEP amplitude at latencies of 30–60 ms (Furubayashi et al. 2000) and probe subcortical excitability (Germann et al. 2023). While the cortex is suppressed at ~70 ms latencies, subcortical neurons are facilitated at 70–80 ms (Tapia et al. 2022), and changes in the magnitude of MEP suppression may indicate altered cortico-reticulospinal interaction (i.e. the balance between cortical suppression and sub-cortical facilitation). These methods may enable non-invasive probing of reticulospinal functioning in humans before and after a training period. While both high force and high rate of force contractions could be expected to lead to reticulospinal adaptation, deliberately slow contractions even to relatively high force levels (~ 75% of maximum) did not show changes in methods purportedly assessing reticulospinal functioning (Akalu et al. 2026). It is important to identify the precise neural adaptation from different forms of resistance training to prescribe targeted interventions for strength performance or rehabilitation better, for example, with an emphasis on examining high force and high rate of force development in isolation.

Therefore, the aim of the present study was to examine corticospinal and reticulospinal functioning following six weeks of either sustained or explosive contraction isometric resistance training, using a randomized controlled trial design. Such training programs have been shown to produce specific adaptations with sustained contractions leading to greater muscle hypertrophy and maximum strength while explosive contractions led to greater muscle activity and rate of force development in the early phase (i.e. < 100 ms) of contraction (Balshaw et al. 2016). The hypothesis proposed that resistance training, by increasing strength and rate of force development, would reduce silent period duration. Second, isometric resistance training would modify the effects of a loud sound so that the StartReact effect would be reduced and the level of MEP suppression would be lower, with explosive resistance training being more efficacious.

## Materials and methods

### Subjects

An a priori sample size calculation was conducted using G*Power 3.1 (Heinrich Heine University of Düsseldorf, Germany) to determine the number of subjects required to detect a significant interaction ((2)time × (3)group) in a repeated measures ANOVA. Meta-analyses have reported an averaged effect size of 0.778 for changes in MEP amplitude following short-term resistance training (Gomez-Feria et al. 2023; Kidgell et al. 2017; Mason et al. 2019; Siddique et al. 2020a). For the present study, the calculation was based on an assumed effect size of *d* = 0.7, α = 0.05, and power (1−β) = 0.95, yielding a required total sample size of n = 36 (i.e. 12 per group). Assuming a 20% drop-out rate, we aimed to recruit ≥15 subjects per group.

**Fig. 1:**
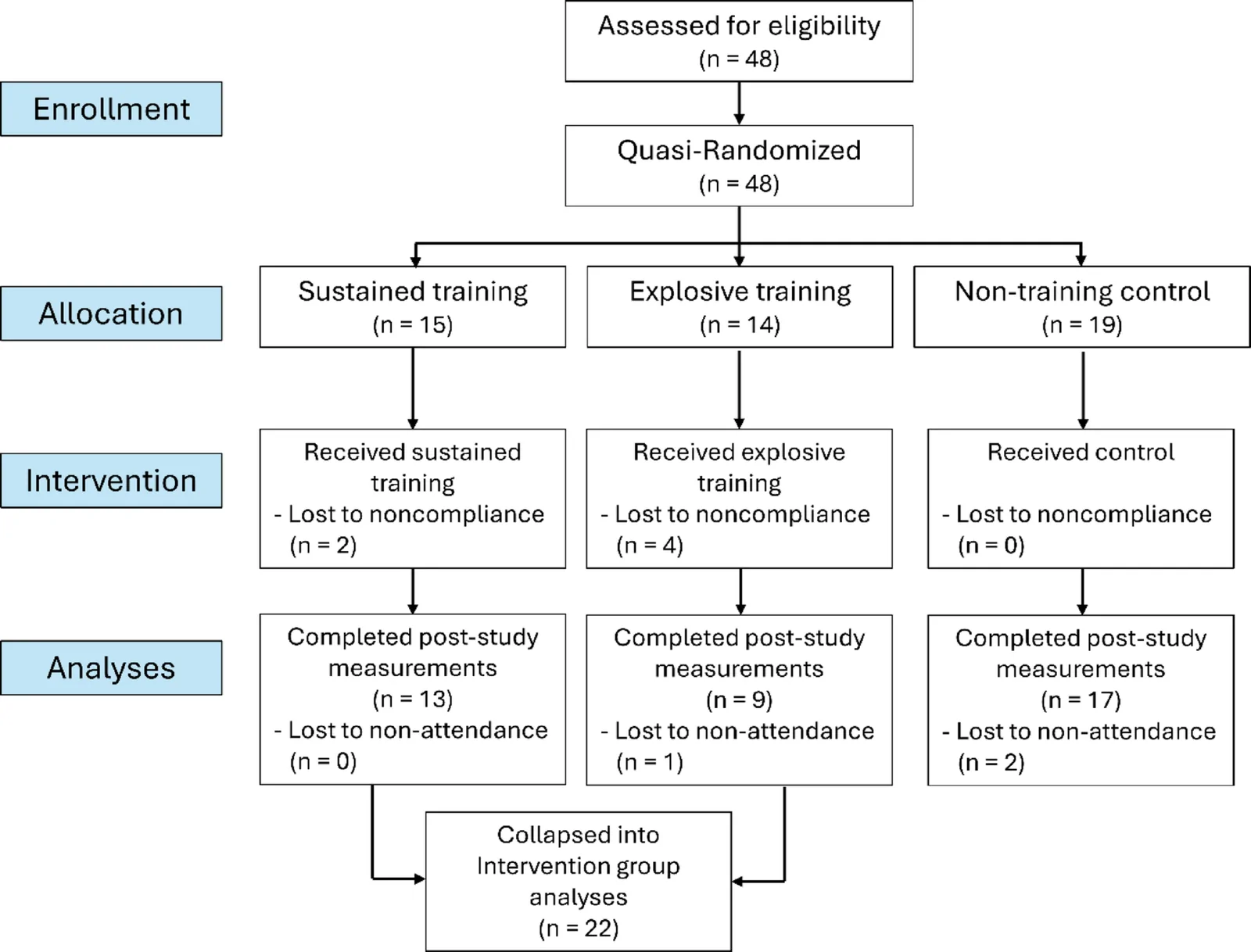
Study flow chart highlighting recruitment, randomization, and follow-up; including collapsing the two training groups into one intervention group

Inclusion criteria were no engagement in resistance training for ≥12 months prior to the study and aged 18–35 years. Volunteers were excluded if they had any medical history that would preclude maximal contractions of the right limb or conditions considered contraindicative for electrophysiological assessments according to Rossi et al. (2021). Forty-eight healthy males volunteered to participate. Recruitment was performed incrementally over a two-year period (2022–2024) due to the number of available portable isometric devices. Once the quota of devices was filled, recruitment was paused until the devices became available. Once suitability for the study had been confirmed and baseline testing performed, volunteers were assigned to one of three groups; (1) sustained contraction training, (2) explosive contraction training, or (3) non-training control. We aimed approximately to match the baseline MVCs of each group. The first 15 subjects were assigned randomly to groups; later in recruitment, group assignment was performed manually with the twin goals of approximately equalizing numbers per group and mean baseline MVC. Recruitment continued until we had fulfilled the necessary estimated sample size for the three-group design. After randomization, there were 15 volunteers selected to sustained training, 14 to explosive training, and 19 to non-training control (Fig. 1).

Nine subjects did not complete the study (7 from the intervention group and 2 from the control group), thus, 39 subjects were included in the final analysis (Sustained training group: age = 21 ± 4 years, height = 176.6 ± 8.3 cm, body mass = 72.9 ± 15.6 kg, BMI = 23.2 ± 3.8 kg/m^2^. Explosive training group: age = 23 ± 5 years, height = 175.0 ± 4.8 cm, body mass = 73.7 ± 23.8 kg, BMI = 23.9 ± 7.3 kg/m^2^. Control group: n = 17 (15 right-handed), age = 24 ± 4 years, height = 176.7 ± 10.3 cm, body mass = 79.2 ± 15.4 kg, BMI = 25.5 ± 5.4 kg/m^2^). Unfortunately, it was not possible to complete enough subjects for the explosive contraction training group to satisfy the estimated statistical power. Thus, as a secondary analysis, the two intervention groups were collapsed into a single group, comprising n = 13 sustained and n = 9 explosive interventions (Intervention group: n = 22 (17 right-handed), age = 22 ± 4 years, height = 176.0 ± 7.0 cm, body mass = 73.2 ± 18.8 kg, BMI = 23.5 ± 5.4 kg/m^2^). Reasons for drop-out were noncompliance with the training program (n = 6) and not able/willing to return for post-study measurements (n = 3).

Subjects provided written informed consent after a verbal explanation of the study procedures, including potential benefits and risks, and were given the opportunity to ask questions. The study was approved by the ethics committee of the Faculty of Medical Sciences at Newcastle University (ethical approval references: 23,483/2022 and 2344/23580). The study was conducted in accordance with the Declaration of Helsinki (2013), except for inclusion in a database.

### Experimental design

**Fig. 2:**
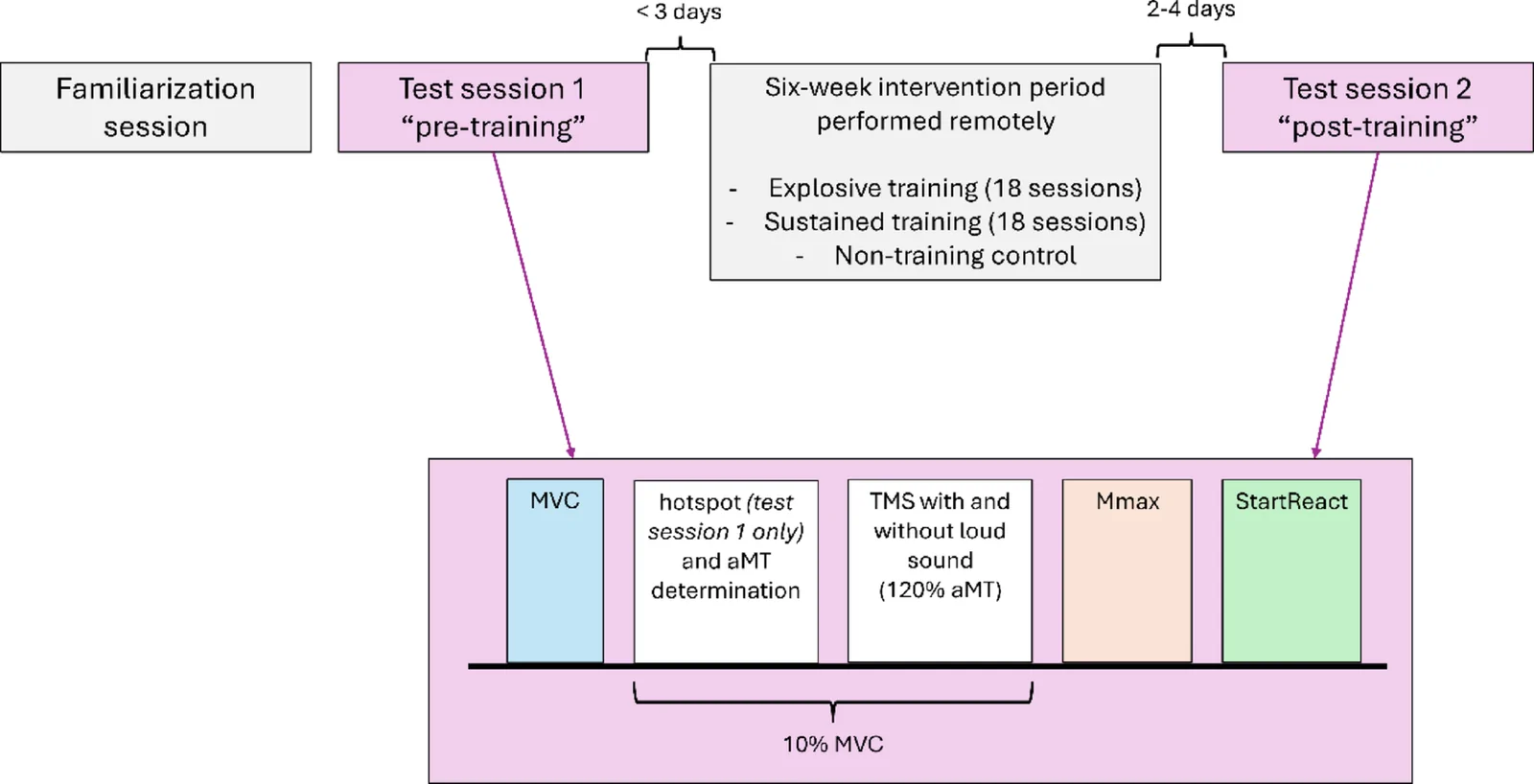
Experimental design showing the three laboratory visits, order of measurements, and home-based isometric resistance training or non-training control period

A quasi-randomized controlled trial design was employed to examine the effect of a short-term resistance training program on the initial neural adaptations of two descending motor pathways, the corticospinal and the reticulospinal tracts. It was aimed to probe adaptations from sustained or explosive contractions within the descending motor pathways, and so a three-group design was performed. Intervention subjects trained three times per week using custom-made isometric dynamometers for a total of eighteen unsupervised sessions while the control group continued their habitual physical activity. TMS was employed to examine the corticospinal and reticulospinal tract without and accompanying auditory stimulation (i.e. loud sound) methods. Subjects attended the laboratory on three occasions. The first visit was a familiarization session, during which subjects reported any relevant medical history or conditions that could exclude them from the study, practiced maximal isometric contractions, and underwent anthropometric measurements (Fig. 2). The second and third laboratory visits occurred immediately before and after the six-week intervention period. In the second visit, subjects in the intervention group were assigned a personal dynamometer, positioned with the elbow at a 90° joint angle. This device configuration enabled standardised, independent training at a joint angle consistent with the testing protocol and associated with maximal elbow flexion force output (Hansen et al. 2003), a practice considered optimal for test–retest reliability. These subjects were tested less than three days before their first training session and within two to four days after their eighteenth session.

### Resistance training intervention

**Fig. 3:**
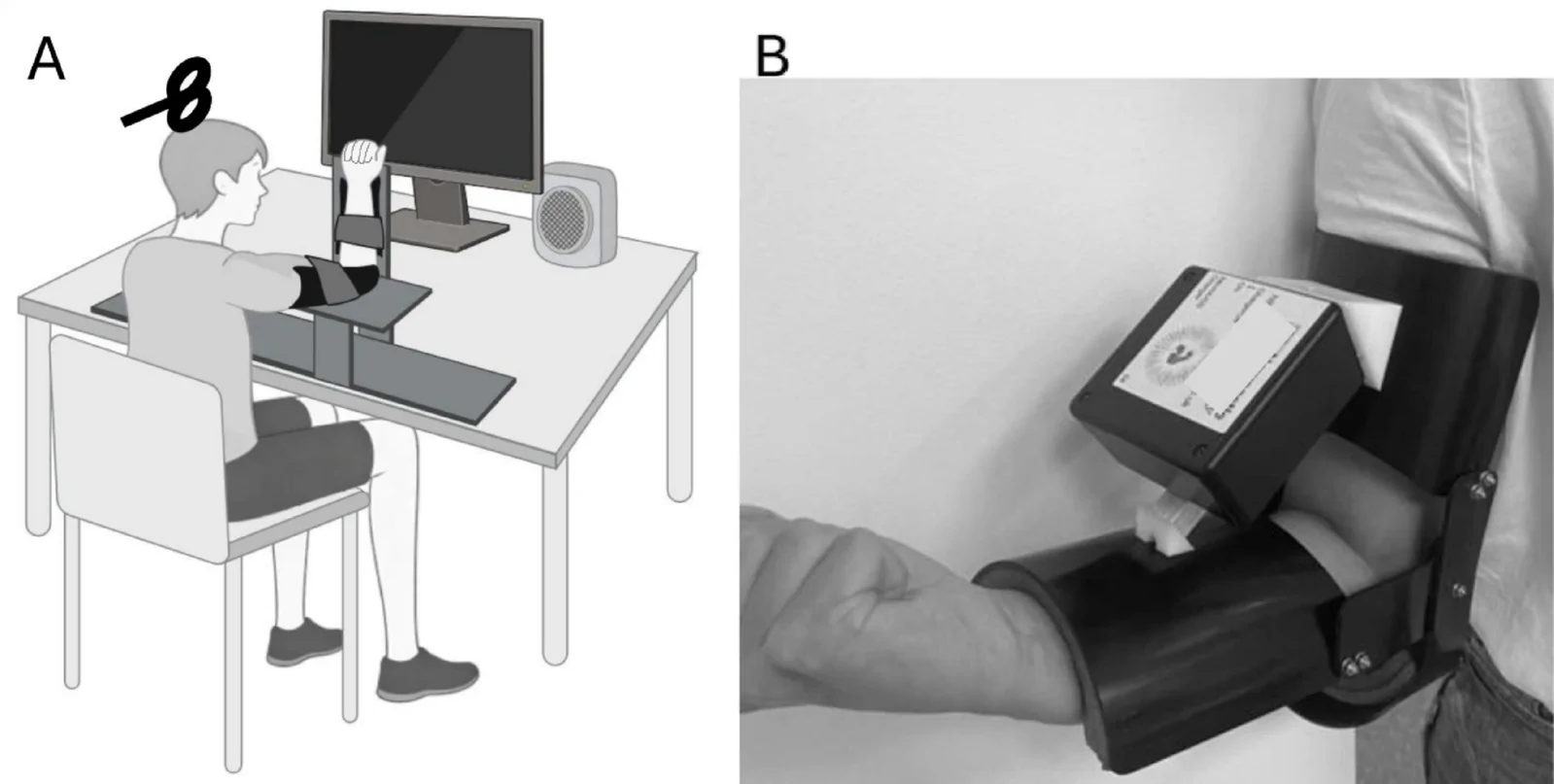
Experimental set up (**A**) showing the isometric dynamometer mounted on a table in front of the subject along with screen, speaker and LED light, and approximate TMS coil position. The portable isometric training dynamometer (**B**). The plastic device was powered by an internal battery in the circuit board housing, which could be recharged through a standard microUSB port. The circuit board connected via Bluetooth to the mobile phone, which in turn connected to the training program website

Intervention group subjects were instructed to perform resistance training three times per week on non-consecutive days at a similar time of day for a total of 18 training sessions. Acceptable training compliance was set at 90%. Subjects performed isometric elbow flexion contractions with their forearm supinated (Fig. 3B). At the start of each session, warm-up contractions were performed comprising three repetitions of sustained contraction at perceived 50%, three repetitions at perceived 70%, and one repetition at perceived 90% of MVC. Four sets of ten repetitions were then carried out with each arm of the main protocol (see below). After each set of ten repetitions, the subject was instructed to change arm. The first training session consisted of four maximal isometric contractions, performed after warm-up and before the four sets of ten repetitions, to set each individual’s target force level for subsequent training sessions. MVC was re-measured on the 5th, 9th, 13th, and 17th session to adjust the target force levels.

The training used a custom-built dynamometer which contained a load cell encased within a housing unit connected to a PVC pipe structure fitted with high-density foam over the forearm and upper arm (Fig. 3B). The device held the elbow joint at 90° and subjects were instructed to maintain a neutral shoulder position with their forearm supinated. The housing on the device held a circuit board which digitized force data and transmitted it over a Bluetooth link to a mobile phone or tablet. Subjects logged onto a website using this device; the website stored their progress through the training program, including the latest MVC performance. Researchers could access training session data stored on the website and track training compliance. The study design aimed to examine possible effects of sustained versus explosive contractions following the methods of Balshaw et al. (2016). Subjects assigned to sustained contractions were required to increase force to 75% of MVC slowly over one second, hold for two seconds and then rest for five seconds between contractions. Explosive contractions were performed as fast as possible and needed to exceed 80% of MVC; these were also followed by five seconds of rest. The website provided real-time force feedback (for sustained group) or rate of force development (for explosive group), together with target force levels, instructions on when to relax and when to contract, and when to change arms.

### Measurements

**EMG:** Following skin preparation, surface electromyography (sEMG) was recorded from the right biceps brachii muscle through bipolar surface electrodes (Kendall H93SG Foam Hydrogel Electrodes, 42 mm × 24 mm, Cardinal Health, USA) placed over the muscle belly (2 cm inter-electrode distance). sEMG signals were amplified and filtered (30–2000 Hz, gain 1000) with a bioamplifier (D360 Amplifier, Digitimer, UK), digitized at a sampling rate of 5 kHz (CED Micro 1401 with Spike2 software, Cambridge Electronic Design, UK) and stored on a computer for off-line analysis.

**MVC:** Subjects were seated with their right arm placed in a dynamometer with non-elastic straps so that the elbow was positioned at shoulder height and the elbow flexed to 90° with palm facing toward the subject (i.e. wrist supinated) (Fig. 3A). A screen was positioned 1 m in front enabling real-time force feedback along with the speaker and light-emitting diode (LED) used in the StartReact and StartleTMS tests. Following a brief warm-up, subjects performed three maximal isometric elbow flexion contractions, separated by 60 s of rest. Subjects were instructed to perform the contraction “as hard as possible” holding the highest force level for ~2 s. The highest force achieved from the three trials was defined as MVC. Thereafter, three more elbow flexions, 60 s apart, were performed “as fast as possible” to determine the highest rate of torque development (RTD). Successful RTD trials were defined as those without an initial drop in force (i.e. countermovement) and exceeding 80% of MVC. Force signals were sampled at 1 kHz, filtered with an acausal Gaussian filter (10 ms SD), and converted from Newtons to Newton-meters (Nm) accounting for the lever-arm distance.

**TMS:** The subjects wore a headband with reflective markers. These were digitized relative to fiducial points on the nasion and the left and right pre-auricular points, allowing head position to be tracked throughout measurements (Polaris Vicra camera, Northern Digital Inc., Waterloo, Canada). The TMS coil was tracked by the same camera allowing for the site of stimulation to be measured using a Brainsight Neuronavigation System (Rogue Research Inc., Montréal, Canada) via the average MNI template.

TMS was applied using a figure-of-eight coil (diameter of each coil 80 mm) through a Magstim 200^2^ magnetic stimulator (The Magstim Company Ltd, Whitland, UK) with a monophasic current waveform. TMS pulses were delivered to induce a posterior-anterior current in the brain. The optimum coil position (‘the hotspot’) to stimulate the left M1 to elicit a MEP in the right biceps brachii was found by holding the coil 45 degrees on the midline moving laterally from the head’s vertex identifying the site of the largest MEP response. This location was found in the first session and saved within the Brainsight system; it was then used for all subsequent sessions. Once this position was found, the active motor threshold was determined as the minimum stimulator intensity to elicit a visible MEP in at least three from six trials. During each stimulation, the subject held a 10% of MVC force level throughout. The contraction was controlled by providing a display of colored bars, which illuminated in sequence as stronger contractions were made. Subjects were asked to maintain the display in the zone indicated by different shades of green bars, which corresponded to 10±1% MVC. The test stimulation intensity was 120% of active motor threshold thereafter. Contralateral MEP area (mV·ms) was quantified and normalized to the maximal compound muscle action potential (Mmax).

**StartleTMS:** A low-intensity auditory cue signaled subjects to contract the elbow flexors and maintain a steady force corresponding to 10% of MVC, as above. Following a randomized delay (5–7.5 s; uniform distribution), TMS was delivered either alone or paired with a loud auditory stimulus (120 dB, 500 Hz, 50 ms duration) presented 50 ms prior to the TMS pulse. Subjects were instructed to relax immediately after stimulation and remain relaxed until the subsequent cue indicating the next contraction. This procedure minimized the development of fatigue. The inter-stimulus interval of 50 ms and the 120 dB auditory stimulus were selected based on previous findings demonstrating that intervals between 30–60 ms and sound intensities greater than 80 dB produce suppression of MEPs (Furubayashi et al. 2000). The protocol consisted of 20 stimuli (10 per condition) delivered in a randomized order, with an inter-trial interval of 22 s. For paired StartleTMS trials, MEP area was expressed as a percentage of the non-paired response.

**StartReact**: A previously validated paradigm known as the StartReact test (Tapia et al. 2022) was used to assess the reticulospinal tract. Reaction time was determined from sEMG onset following three stimulus conditions: a visual cue alone (visual reaction time; VRT), a visual cue paired with a quiet auditory stimulus (visual–auditory reaction time; VART; 80 dB, 500 Hz, 50 ms), and a visual cue paired with a loud auditory stimulus (visual–startle reaction time; VSRT; 120 dB, 500 Hz, 50 ms). Visual and auditory stimuli were delivered simultaneously. The difference in reaction time between VART and VSRT reflects activation of reticular formation pathways (Tapia et al. 2022) and is termed the StartReact effect (SReffect). Subjects were seated with the right forearm supinated and resting on the right quadriceps. They were instructed to flex the elbow rapidly, moving their hand toward the shoulder when a red LED positioned approximately 1 m in front of the subject was illuminated. Reaction time was defined as the interval between LED onset and the initiation of the sEMG burst. The three stimulus conditions were presented in randomized order with 20 trials per condition and a 10 s inter-trial interval.

**Mmax assessment:** With the arm remaining in the force transducer (Fig. 3A) but in a relaxed state, the anode was placed on the supraclavicular fossa (i.e. Erb’s point) and the cathode on the acromion process (Kendall H34SG ECG Foam Hydrogel Electrodes, 50 mm × 45 mm, Cardinal Health, USA). A constant current isolated electrical stimulator (DS7AH, Digitimer LTD, UK) was used. Current intensity was progressively increased by 10 mA with stimuli every five seconds until the maximal sEMG response in biceps brachii was reached (i.e. when the M-wave peak-to-peak amplitude saturated). Thereafter, 125% of maximal response intensity was used to elicit three separate twitches from which the highest Mmax area (mV·ms) and twitch torque (Nm) values were taken.

### Data analysis

Data analyses were performed using customized scripts in MATLAB R2024b software (The MathWorks, Inc., Natick, Massachusetts, USA). sEMG signals were averaged for all trials, full wave rectified, visualized, and the average pre-stimulus sEMG activity level highlighted. The investigator manually assigned the onset and offset of the MEP as well as the end of the silent period based on visual inspection of the sEMG signal relative to baseline (i.e. average sEMG amplitude 200 ms prior to the stimulus). The MEP onset was defined as the first deflection 2 SD above the average rectified pre-stimulus sEMG activity and the offset defined as the point where the average rectified waveform falls below the pre-stimulus level. Contralateral MEP area was, thus, the rectified area above the average rectified pre-stimulus activity. MEP area (mV·ms) was then normalized as a percentage of Mmax area (mV·ms) to provide a measure of the fraction of the muscle that was recruited. This is a common procedure to account for possible changes in the tissue-electrode interface (Lanza et al. 2018), e.g. electrode location, skin preparation and impedance, subcutaneous fat, muscle mass, between sessions. The stimulation trigger, and MEP onset and offset time-points were used to establish MEP latency and duration (ms). Contralateral silent period duration (ms) was defined as the time from the stimulation until the return of the average rectified sEMG activity to baseline.

Reaction times from the StartReact test were determined as the moment when the rectified sEMG surpassed seven standard deviations (SD) above the average EMG activity measured from 0 to 200 ms before the stimulus onset (Germann and Baker 2021). Visual inspection of each trial was conducted using a custom MATLAB script, which automatically identified the previously defined threshold for onset. Any inaccurate onset detections, possibly due to electrical noise artifacts, were manually adjusted. This process resulted in a reaction time distribution for each condition.

For RTD analysis, the maximum instantaneous force produced during the contraction (defined as MVC) was identified, and then contraction onset was determined at the point where 1.5% of MVC was reached. Contraction onset was visually verified and modified if necessary. RTD over 0–50 ms was obtained to gain insight into neural adaptations, which purportedly drive the early phase (< 75 ms) of rapid force development (Maffiuletti et al. 2016). The highest MVC and RTD values were used for statistical analysis. Reliability between the familiarization and pre-training sessions was ICC = 0.872 with a standard error of the mean (SEM) of 36 Nm for MVC, ICC = 0.675 with a SEM of 367 Nm·s⁻^1^ for RTD, and ICC = 0.500 with a SEM of 4.7 Nm for twitch torque.

### Statistical analysis

All analyses were performed with IBM SPSS Statistics version 28 software (SPSS, Inc., Chicago, Illinois, USA). Normality was assessed by the Shapiro–Wilk test. Any variables that were not normally distributed (Age, MEP area, MEP latency, and MEP duration) were transformed to a logarithmic scale (log_10_) prior to further statistical tests, but the data are presented as uncorrected values. To determine the effects of the intervention, all variables were assessed using repeated measures ANOVA ((2) time × (3) group). Any significant main effect was evaluated by pairwise comparisons using Bonferroni’s adjustments. Similarly, for the secondary analyses, repeated measures ANOVA ((2) time × (2) group) with Bonferroni adjustments were performed. Direct, between-group comparisons, i.e. for baseline characteristic comparisons, were performed using independent T-tests. Partial eta squared effect sizes were transformed to Cohen’s *d* (Cohen’s *f* = √ η^2^_p_ / 1−η^2^_p_, Cohen’s *d* = 2 *f*), where < 0.2 denotes small, 0.2–0.8 denotes medium, and > 0.8 denotes large effects. Data are presented as the non-transformed mean ± SD. Alpha was set as 0.05.

## Results

**Fig. 4:**
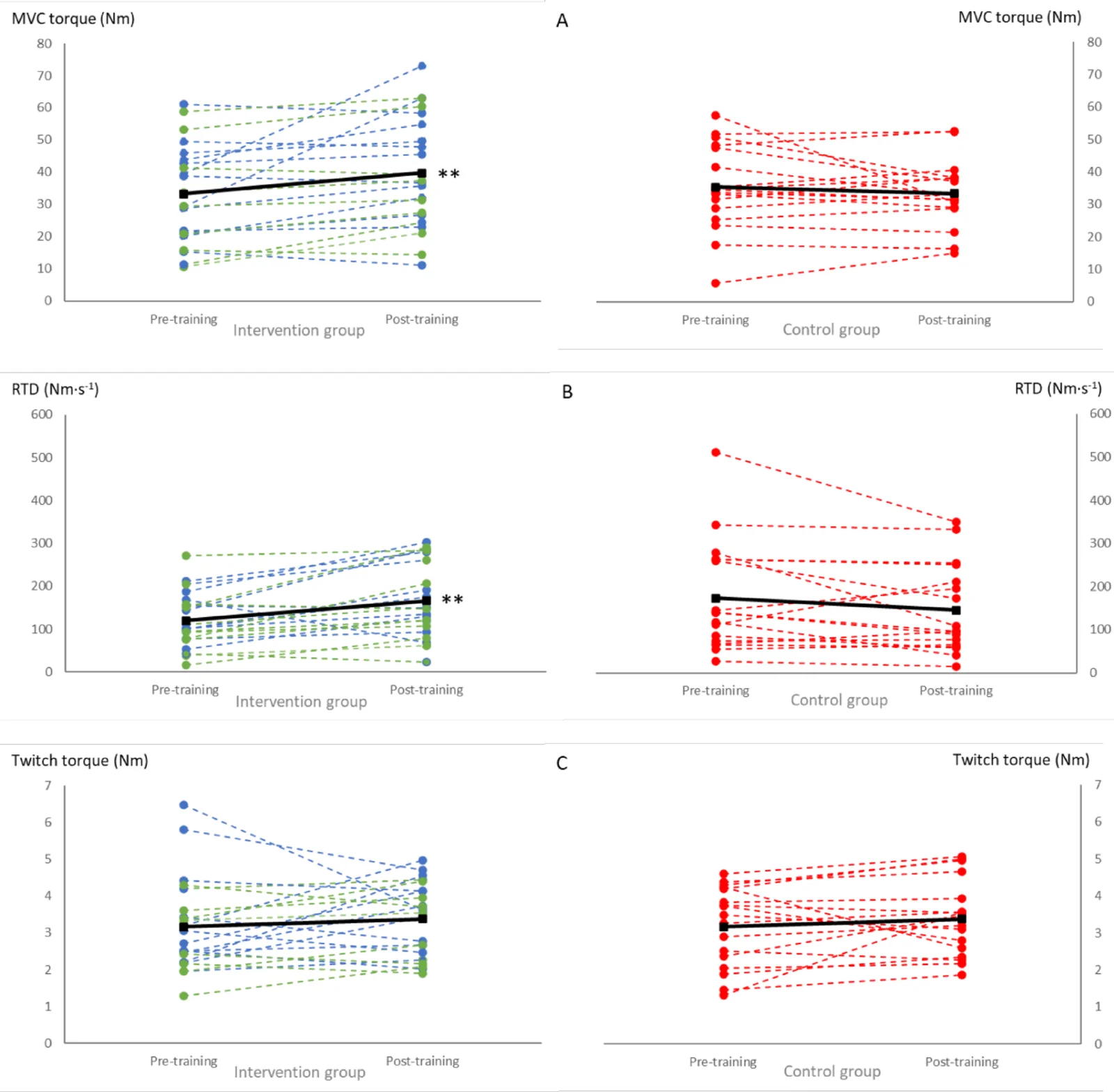
Voluntary and stimulus-evoked isometric torque before and after the six-week period in intervention and control groups. Individual data are shown by hashed lines (sustained training group = blue, explosive training group = green, control group = red) while mean data are shown by solid black lines. Pre- to post-training statistical significance (P < 0.01) is denoted by **

There were no differences in subject characteristics (i.e. age, height, body mass, BMI) nor in MVC, RTD or twitch torque between the intervention and control groups at baseline. Significant time × group interactions were observed for MVC (F_2,36_ = 3.94, P = 0.028) and RTD (F_2,36_ = 6.65, P = 0.003) from the three-group comparison. Post hoc tests revealed that only the sustained training group significantly increased MVC (P = 0.007, *d* = 0.96) after training, whereas both the sustained and explosive training groups increased RTD (P = 0.009, *d* = 0.92, P = 0.045, *d* = 0.69, respectively) after training (Table 1). When the intervention groups were collapsed, there was a significant time × group interaction for MVC (F_**1,37**_ = 7.53, P = 0.009) and RTD (F_**1,37**_ = 13.64, P < 0.001) with the intervention group increasing MVC (P = 0.003, *d* = 1.06) and RTD (P = 0.003, *d* = 1.15) significantly over the six-week period (Fig. 4). No main effects were observed for twitch torque or Mmax.

**Table 1:** Outcome measures (mean ± SD) for the sustained (n = 13) and explosive (n = 9) isometric resistance training and non-training control (n = 17) groups

	MVC (Nm)	RFD (Nm/s)	Twitch torque (Nm)	SReffect (ms)	MEP area (% Mmax area)	MEP duration (ms)	Silent period (ms)	StartleTMS (% of non-paired responses)
*Sustained training group*								
Pre-study	35.2 ± 13.6	131.6 ± 50.4	3.39 ± 1.44	28.0 ± 14.8	10.5 ± 19.2	15.6 ± 4.4	84.2 ± 18.8	73.9 ± 39.4
Post-study	42.9 ± 17.4*	180.4 ± 78.2*	3.65 ± 0.97	18.2 ± 15.7*	3.4 ± 3.2*	15.0 ± 5.4	54.1 ± 32.8*	34.0 ± 40.0*
Δ%	24.2 ± 40.3	44.7 ± 50.6	18.7 ± 42.9	− 28.3 ± 73.3	− 48.2 ± 38.4	− 3.1 ± 21.1	− 30.9 ± 47.2	− 45.7 ± 55.8
*Explosive training group*								
Pre-study	30.6 ± 17.8	102.6 ± 79.5	2.84 ± 0.95	10.6 ± 11.7	7.2 ± 5.2	14.8 ± 2.9	81.6 ± 24.1	86.3 ± 40.8
Post-study	35.4 ± 16.8	146.6 ± 95.2*	2.99 ± 0.93	10.6 ± 9.8	3.0 ± 2.2*	12.3 ± 2.3	69.6 ± 19.1	104.4 ± 59.6
Δ%	30.0 ± 45.5	80.8 ± 130.0	9.7 ± 27.6	− 10.8 ± 94.4	− 44.0 ± 44.0	− 14.7 ± 19.8	− 10.4 ± 22.8	28.2 ± 47.6
*Control group*								
Pre-study	35.3 ± 13.4	173.2 ± 128.2	3.19 ± 1.08	23.2 ± 12.2	19.5 ± 28.4	14.9 ± 2.4	84.1 ± 30.0	73.7 ± 62.5
Post-study	33.3 ± 10.2	145.4 ± 103.0	3.42 ± 1.03	18.4 ± 10.7	15.0 ± 26.1	14.4 ± 1.9	70.9 ± 30.5	71.1 ± 61.6
Δ%	5.5 ± 44.9	− 9.5 ± 39.7	15.2 ± 44.6	− 7.8 ± 55.0	4.8 ± 113.1	− 2.2 ± 14.4	− 14.8 ± 26.2	33.1 ± 116.7

^*^ = P < 0.05 for within-group difference between pre- and post-study measurements

**Fig. 5:**
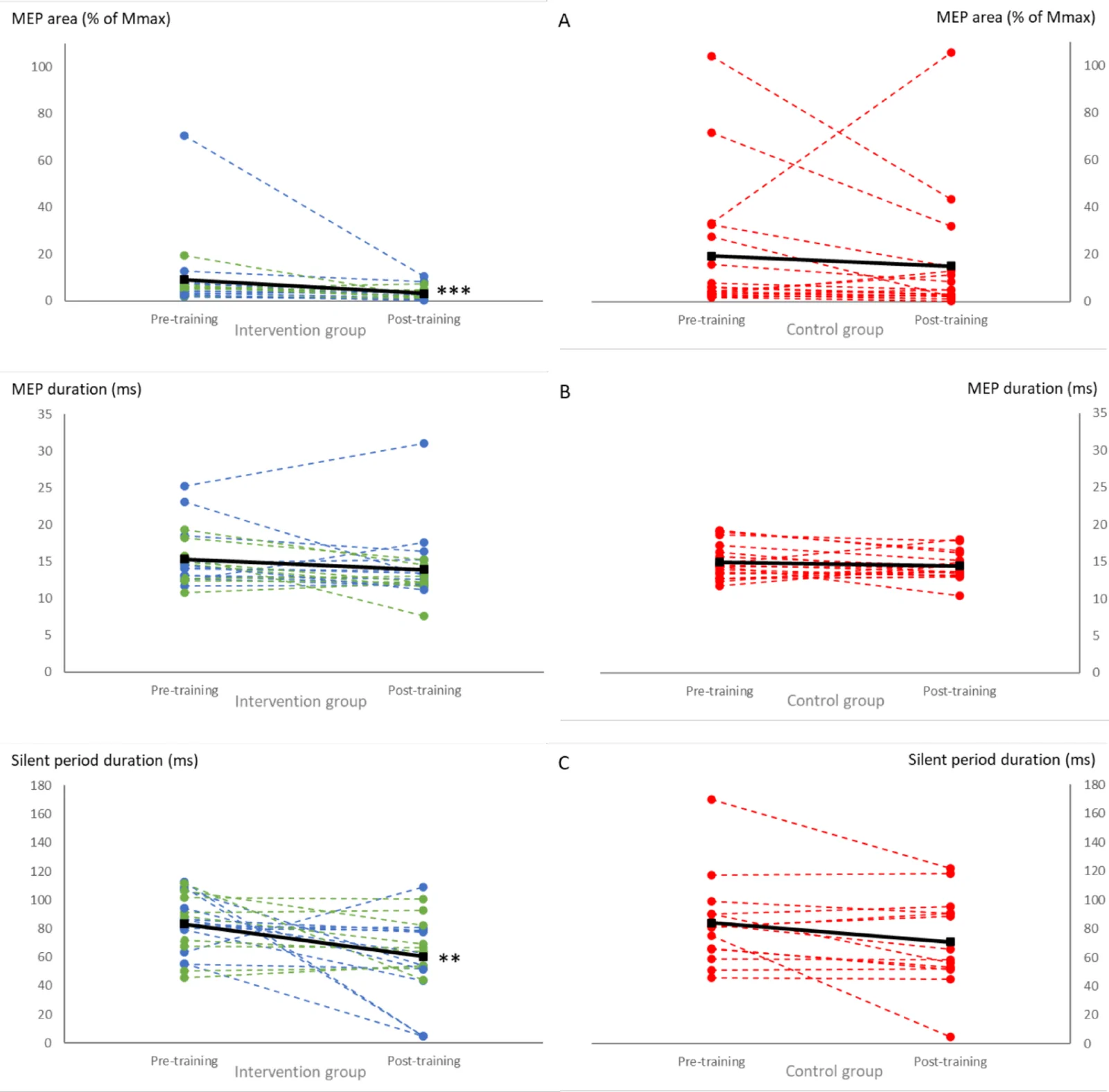
MEP responses to TMS alone before and after the six-week period in intervention and control groups. Individual data are shown by hashed lines (sustained training group = blue, explosive training group = green, control group = red) while mean data are shown by solid black lines. Pre- to post-training statistical significance (P < 0.01) is denoted by ** and (P < 0.001) by ***. The effects of possible outliers were explored statistically but their removal did not modify the observed effects; MEP area remained different (P < 0.01) as well as silent period duration (P = 0.025) in the intervention groups. Therefore, all data are included and presented

There were no significant time × group interactions for any MEP variable from the three-group comparisons nor when the intervention groups were collapsed. However, significant main effects of time were observed for MEP area normalized to Mmax area (F_1,34_ = 22.17, P < 0.001) and silent period duration (F_1,35_ = 10.04, P = 0.003) from the three-group comparison. This was also observed when the intervention groups were collapsed (F_1,35_ = 19.74, P < 0.001 and F_1,36_ = 8.87, P = 0.005, respectively). Post hoc tests revealed that both sustained and explosive training groups decreased MEP area (P = 0.002, *d* = 1.18 and P = 0.009, *d* = 0.96, respectively, Table 1), which carried over when the intervention groups were collapsed (P < 0.001, *d* = 1.42, Fig. 5A). No main effects were observed in MEP duration (Fig. 5B). Silent period duration was significantly reduced in the sustained training group only (P = 0.002, d = 1.13; Table 1) and when the intervention groups were collapsed (P = 0.002, d = 1.08; Fig. 5C).

**Fig. 6:**
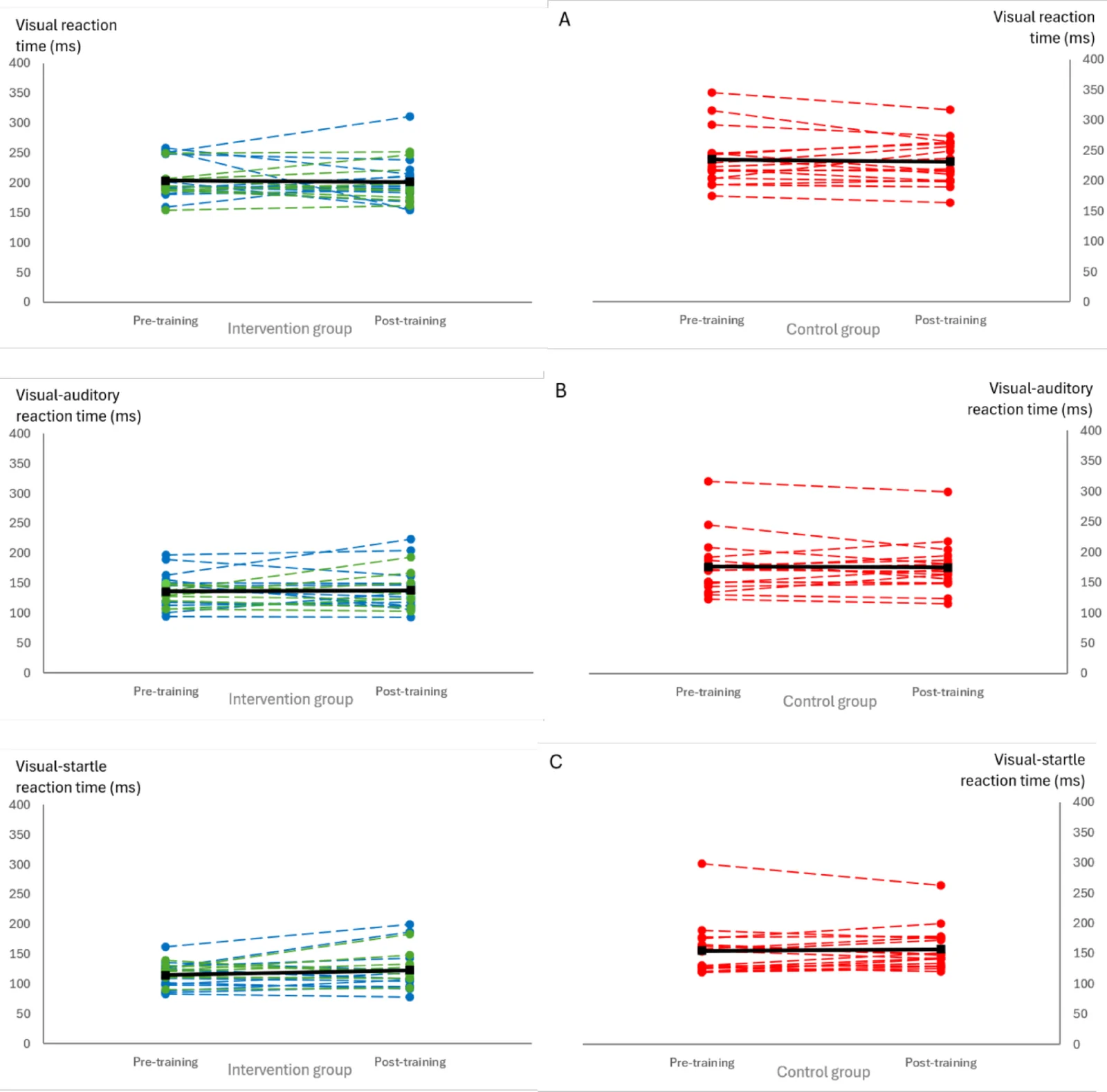
Time (ms) from the presentation of the light to the onset of the EMG response during the StartReact test before and after the six-week period in intervention and control groups. Reaction time to a visual cue alone (VRT), a visual cue paired with a quiet auditory stimulus (VART) and a visual cue paired with a loud auditory stimulus (VSRT) are shown. Individual data are shown by hashed lines (sustained training group = blue, explosive training group = green, control group = red) while mean data are shown by solid black lines

Figure 6 shows the reaction times from the StartReact test. There was no significant time × group interaction for the StartReact effect (VART–VSRT) for the three-group comparison, but there was a main effect of time (F_1,34_ = 4.29, P = 0.046). Here, the sustained training group showed significant reductions over the six-week period (P = 0.016, *d* = 0.87, Table 1). When the intervention groups were collapsed, a main effect of time was observed (F_1,35_ = 4.88, P = 0.034) but there were no post hoc differences over time.

There was a significant time × group interaction for the suppression in MEP area due to StartleTMS (F_1,33_ = 4.67, P = 0.016) with a significantly greater suppression in the sustained training group (P = 0.004, *d* = 1.08, Table 1). This was not observed when the intervention groups were collapsed.

## Discussion

The present study investigated the potential impact of a six-week isometric resistance training intervention on corticospinal and reticulospinal functioning. Although the explosive training group had fewer subjects than required to satisfy a priori sample size for MEP amplitude, some evidence of neurophysiological adaptation can be drawn from the present study. Results showed that both maximal and rapid isometric force production was enhanced through training. Accompanying these functional changes, MEP area (both training groups) and silent period duration (sustained group only) decreased. Reduced silent period duration, putatively indicative of reduced intracortical inhibition, agrees with the study hypothesis. Regarding potential reticulospinal adaptation, the results demonstrate conflicting findings for the sustained training group. While reaction time enhancement from loud sound was reduced, suggesting reduced reliance on the sound to induce subcortical activation, the level of MEP suppression by a loud sound increased after training. Thus, the second hypothesis could not be confirmed.

As expected, the short-term resistance training program led to increases in both maximum strength and RTD (0–50 ms), with the changes being statistically greater than those demonstrated by the control group. The magnitude of improvement in isometric force measures (MVC = approx. 27%, RTD = approx. 60%) is somewhat larger than previously observed from isoinertial training over a similar training duration (e.g. ~15%, Gomez-Guerrero et al. 2024; ~12–20%, Häkkinen and Komi 1981). This is likely due to the isometric training mode (Folland et al. 2005) and could be also influenced by the untrained state of the subjects. While strength, and its improvement, is influenced by muscle mass/hypertrophy (Maughan et al. 1984), RTD over the initial 50 ms of contraction is thought to be governed entirely by neural factors (Maffiuletti et al. 2016). Given the lack of increase in twitch torque, an indirect measure of muscle mass (e.g. Behrens et al. 2016), it may be assumed that most, if not all, of the strength increase observed was due to enhanced neural activation. In this regard, the remote and online isometric training intervention was successful.

Meta-analyses have suggested that the most robust adaptation to short-term resistance training observable in electrophysiological measures is a reduction in cortical inhibition (Kidgell et al. 2017; Siddique et al. 2020a). Both silent period duration, purportedly representing γ-aminobutyric acid (GABA-_B_) neurotransmission, and short-interval intracortical inhibition, purportedly representing GABA-_A_ neurotransmission, are reduced following resistance training (Kidgell et al. 2017; Siddique et al. 2020a). In the present study, silent period duration demonstrated reductions after six weeks of sustained training with large effect sizes (*d* > 1) from both forms of isometric training, suggesting reduced inhibition.

Similarly, the present study observed significantly reduced MEP area in both training groups whereas no within-group change was observed in the control group. It might have been expected that MEP size would increase after resistance training, as is predominantly observed (Kidgell et al. 2017; Siddique et al. 2020a), due to enhanced cortical excitability and/or transsynaptic corticospinal propagation (Siebner et al. 2022). Indeed, it has been shown that four weeks of externally paced dynamic resistance training increased corticospinal excitability whereas isometric training led to no change (Siddique et al. 2020b). Nevertheless, some previous studies have observed similar reductions (Gomez-Guerrero et al. 2024; Lee et al. 2009). The corticospinal tract is specialized for force control and fine tuning (Maier et al. 1993), but the isometric force test/training used in the present study did not require advanced limb coordination nor did it rely heavily on proprioceptive feedback. Therefore, it may be that the cortical/corticospinal excitation to initiate muscle contraction was already more than sufficient, and perhaps overly excitable (indicative of hyperactivity of pyramidal neurons and inefficiency). It may be that generic cortical neuronal discharge is sufficient since similar pyramidal activity has been shown to occur irrespective of required force level in monkeys (Glover and Baker 2022). Nevertheless, the precise cause of the reduced MEP area after resistance training remains unresolved.

Loud sounds have been shown to facilitate output from the reticulospinal tract, while inhibiting neurons in the cortex over a short period (e.g. 30–60 ms) (Furubayashi et al. 2000; Tapia et al. 2022). Therefore, the StartReact test, which examines the difference in reaction time to visual stimuli accompanying different sound conditions, can reveal reticulospinal efficacy. Tapia et al. (2022) suggested that an increased contribution from the reticulospinal tract would result in increased StartReact effect (i.e. a greater difference between sEMG reaction times to loud vs quiet auditory cues). In our previous cross-sectional study, examining long-term strength-trained individuals versus untrained controls, the StartReact effect was lower in the trained athletes than the untrained subjects (Tanel et al. 2025). We suggested this to be because the reticulospinal contribution had become so large that it was already close-to-maximal in strength-trained athletes during the quiet auditory cue condition. Based on this information, the present study hypothesized that six weeks of training would enhance reticulospinal function, thereby reducing the StartReact effect (i.e. a reduced difference between sEMG reaction times to loud vs quiet auditory cues). Here, a decrease in StartReact effect occurred in the sustained training group (approx. –28%, P = 0.004) but not the explosive training group nor when the groups were collapsed. It may be speculated that the adaptation was training protocol-specific but given the small sample size of the explosive training group, such an interpretation is open to type II error. The absence of significant changes in the present study may be related to the sensitivity of the StartReact effect. As reported by Tapia et al. (2022), a ceiling effect may occur when input from the reticular formation exceeds ~80% of the total drive, leaving less than ~20% contribution from M1 and limiting the ability to detect further changes.

TMS using a 50 ms latency from loud sound to magnetic stimulation (i.e. StartleTMS) tends to lead to a suppression of MEP size on a group level (Furubayashi et al. 2000). However, some individuals demonstrate facilitation (Germann and Baker 2021; Tanel et al. 2025), as highlighted here by the large SD values. One previous study also failed to identify significant differences between long-term strength-trained athletes versus non-trained controls (Tanel et al. 2025), although there was a tendency towards lower suppression/higher facilitation in the trained group. As previously noted, the 50 ms latency seems to induce both facilitation (from subcortical sources) and inhibition (from cortical sources), and the final measure is the result of a trade-off between these two opposing influences. Thus, it is not known whether the significantly greater suppression observed in the sustained training group was due to increased cortical inhibition or reduced subcortical facilitation or both. Speculatively, the reduced cortical inhibition during low-force voluntary contraction after training may have created the potential for the loud sound to induce a higher level of cortical inhibition. Nonetheless, greater StartleTMS suppression does not support the hypothesis that reticulospinal function increases with resistance training and is in opposition to our interpretation of our StartReact results.

### Study limitations

This was an unsupervised, at-home isometric training program. While the software documented the training to track compliance, the program may not have been optimal to adjust the required force level or the subject may have not been accurate in attaching the device to their arm as instructed, which may have affected reproducibility, suitability and consequent maximal strength improvement. Further, a lack of on-site encouragement and supervision may have affected the subjects’ performance during training. Nevertheless, these methods may also be considered a strength of the study since the training and testing method were both isometric and performed at the same joint-angle. Such specificity is typically lacking in training intervention studies.

The number of subjects that completed the explosive resistance training intervention were fewer than required to satisfy the estimated statistical power and the sustained training group also had <15 subjects. Therefore, to enhance statistical power and test our hypotheses more robustly, we collapsed the two intervention groups. However, this served to nullify the observed changes observed from StartReact and StartleTMS in the sustained training group. It may be that explosive training led to different adaptations than sustained training, but we cannot discount statistical error. Ultimately, it should be considered that the effect of explosive resistance training on corticospinal and reticulospinal functioning remains unresolved.

MEP responses were only tested at 120% of active motor threshold. Low stimulation intensity primarily, if not exclusively, excites early I-waves indicative of more direct motor pathways. Higher stimulation intensities are likely needed to exert influence from poly- and/or oligosynaptic pathways, such as the subcortical reticulospinal tract. Indeed, MEP area differences between long-term strength trained athletes and controls were only observed at the highest stimulation intensities (Tanel et al. 2025). Therefore, it is recommended to test the full recruitment curve in future.

Additionally, examining the bilateral reticulospinal tract may be possible through ipsilateral MEPs. We have recently shown that it is possible to induce robust iMEPs using dynamic bilateral elbow flexion actions (Maitland and Baker 2021; Hu et al. 2024). While such testing adds complexity to the test battery, it may provide useful information and overcome the difficulty in assigning the source of adaptation (i.e. cortex suppression or subcortex facilitation or both) with using loud sound.

Finally, only male subjects were tested in the present study. Fluctuations in female sex hormones across the menstrual cycle may modulate cortical/corticospinal excitability (Smith et al. 2002); however, the extent and consistency of these effects remain under investigation. Controlling for menstrual cycle phase remains recommended and requires tracking menstrual status, confirming hormone concentrations, and scheduling measurements accordingly. As these procedures were beyond the resources of the present study, only male subjects were tested. This limitation restricts the generalisability of the findings to males.

In conclusion, short-term unsupervised isometric resistance training led to robust increases in strength in previously untrained adults. The strength improvements were accompanied by reduced MEP area and reduced silent period duration, but conflicting data between the StartReact effect and the magnitude of MEP suppression following loud sound in the isometric training group. These findings suggest neuroplastic adaptations within M1 and/or corticospinal tract. However, the direction of changes in measures purported to reflect reticulospinal functioning complicates interpretation. Consequently, current research focuses on both the corticospinal and reticulospinal pathways as potential substrates of neural adaptations to resistance training. Although indirect assessment of subcortical pathways in humans remains challenging, more refined and targeted methodological approaches are needed to provide clearer evidence.

## Data Availability

The data used in this article is freely available from the Mendeley Data Repository (https://data.mendeley.com/research-data/) from 1.10.2026 https://doi.org/10.17632/f5hc9nfmbz.1
